# Cortical tau is associated with microstructural imaging biomarkers of neurite density and dendritic complexity in Alzheimer's disease

**DOI:** 10.1002/alz.13011

**Published:** 2023-03-18

**Authors:** Philip S. J. Weston, William Coath, Matthew J. Harris, Ian B. Malone, John Dickson, Franklin I. Aigbirhio, David M. Cash, Hui Zhang, Jonathan M. Schott

**Affiliations:** ^1^ The Dementia Research Centre, Department of Neurodegenerative Disease UCL Queen Square Institute of Neurology London UK; ^2^ UK Dementia Research Institute at UCL University College London London UK; ^3^ Institute of Nuclear Medicine University College London Hospitals London UK; ^4^ Department of Clinical Neurosciences University of Cambridge Cambridge UK; ^5^ Wolfson Brain Imaging Centre University of Cambridge Cambridge UK; ^6^ Department of Computer Science and Centre for Medical Image Computing University College London London UK

**Keywords:** Alzheimer's disease, cerebral cortex, diffusion MRI, microstructure, neurite orientation dispersion and density imaging, positron emission tomography, tau

## Abstract

**Introduction:**

In Alzheimer's disease (AD), hyperphosphorylated tau is closely associated with focal neurodegeneration, but the mechanism remains uncertain.

**Methods:**

We quantified cortical microstructure using neurite orientation dispersion and density imaging in 14 individuals with young onset AD. Diffusion tensor imaging measured mean diffusivity (MD). Amyloid beta and tau positron emission tomography were acquired and associations with microstructural measures were assessed.

**Results:**

When regional volume was adjusted for, in the medial temporal lobe there was a significant negative association between neurite density and tau (partial R^2^ = 0.56, *p* = 0.008) and between orientation dispersion and tau (partial R^2^ = 0.66, *p* = 0.002), but not between MD and tau. In a wider cortical composite, there was an association between orientation dispersion and tau (partial R^2^ = 0.43, *p* = 0.030), but not between other measures and tau.

**Discussion:**

Our findings are consistent with tau causing first dendritic pruning (reducing dispersion/complexity) followed by neuronal loss. Advanced magnetic resonance imaging (MRI) microstructural measures have the potential to provide information relating to underlying tau deposition.

## 1 INTRODUCTION

Alzheimer's disease (AD) is the canonical cortical dementia, with deposition of molecular pathology followed by progressive cortical neuronal loss and cognitive decline. The deposition of hyperphosphorylated tau, which follows amyloid beta (Aβ), begins in most cases in the medial temporal lobe (MTL), before propagating through the neocortex as disease progresses.^1^ A close topographical association exists between tau and neurodegeneration,^2^ although the mechanism by which tau causes neuronal dysfunction and loss remains uncertain. Histopathology findings suggest an association between tau and dendritic morphology; however, in vivo confirmation is lacking.^3^ The development of tau positron emission topography (PET) allows in vivo quantification of cortical tau.^2^

Magnetic resonance imaging (MRI) methods for automated measurement of cortical thickness and/or volume allow the detection of macrostructural loss.^4^ More recently, diffusion‐weighted MRI (dMRI), primarily using diffusion tensor imaging (DTI), has enabled the assessment of cortical changes at the microstructural level, usually through the metric of mean diffusivity (MD).^5^, ^6^ However, DTI provides only a composite view of each voxel, so cannot distinguish the characteristics of dendritic trees from the surrounding cellular structures or cerebrospinal fluid (CSF).^7^ It is, therefore, unclear to what extent the cortical MD signal relates to CSF partial volume due to macrostructural atrophy versus changes in neuronal microstructure. The development of multi‐shell dMRI, using multiple diffusion weightings, allows for multi‐compartmental modeling, with the most widely used and validated being neurite orientation dispersion and density imaging (NODDI).^8^, ^9^ NODDI models neural tissue as separate tissue‐specific compartments within the same voxel. It becomes possible to explore specific disease‐related processes, including estimating both the orientation dispersion index (ODI)—providing information relating to dendritic complexity—and the neurite density index (NDI), with both measures having undergone histological validation.^9^, ^10^ We would hypothesize that as neurodegeneration progresses, dendrites, which are the predominant neurite in cortical gray matter, would reduce in density and structural complexity, causing a decrease in both NDI and ODI measures.

Here we used Aβ PET, tau PET, and multimodal MRI, including NODDI, in individuals with young onset AD (age at onset <65), capitalizing on the more variable clinical phenotype in younger patients. We explore associations between molecular pathology and neuronal/dendritic microstructure, and compare with more conventional volume and DTI metrics.

## 2 MATERIALS AND METHODS

Fourteen participants were recruited from the Cognitive Disorders Clinic at the National Hospital for Neurology and Neurosurgery. All met the diagnostic criteria for AD,^11^ with an even mixture of typical amnestic and posterior cortical atrophy (PCA) phenotypes. Cognition was assessed using the Addenbrookes Cognitive Examination (ACE) III.^12^

**RESEARCH IN CONTEXT**

1. **Systematic Review**: Previous studies of tau positron emission tomography (PET) with cortical magnetic resonance imaging (MRI) generally used brain volume/thickness, which cannot differentiate specific aspects of neurodegeneration. Although the pathological link between tau and neurodegeneration has been investigated in animal models of Alzheimer's disease (AD), including with neurite orientation dispersion and density imaging (NODDI), this has not extended to human studies.
2. **Interpretation**: We demonstrate that specific in vivo measures of tissue microstructure are closely linked to focal cortical tau, supportive of the hypothesis, derived from mouse studies, that tau causes first dendritic pruning followed by loss of neurite density. NODDI, acquired on standard clinical MRI scanners, may therefore provide sensitive and specific information relating to underlying tau pathology.
3. **Future Directions**: It will be important to assess the association between tau and NODDI measures identified in the current study in larger cohorts, including at earlier stages of disease. In addition, longitudinal study will be informative in confirming the temporal sequence of molecular and microstructural changes.

Aβ PET was acquired on a 3T Biograph mMR PET‐MRI scanner (Siemens Healthcare), with injection of 370 MBq of [18F]Florbetapir, using the acquisition and processing methods described previously, with a positive SUVR cutoff of >0.618.^13^, ^14^tau PET was undertaken on a PET‐CT (computed tomography) scanner, with static image acquisition at 70–80 min after injection of 370 MBq of [18F]Flortaucipir. Standardised uptake value ratios (SUVRs) were calculated using an inferior cerebellar reference region.

MRI was acquired concurrently during the Aβ‐PET scan. Acquisitions included a Magentization‐Prepared Rapid Acquisition Gradient Echo (MPRAGE, (repetition time [TR] = 2000 ms, echo time [TE] = 2.92 ms, voxel size 1.1 mm^3^) and multi‐shell dMRI (TR = 8000 ms, TE = 103 ms, voxel size 2.5 mm^3^), including *b*‐values of 2000 (64 directions); 700 (32 directions) and 0 s/mm^2^ (12 repetitions). Cortical gray matter parcellation of T_1_‐weighted images was performed using GIFv3,^15^ with subsequent resampling of the GIF‐parcellated regions into amyloid‐PET, tau‐PET, and dMRI space to enable region of interest (ROI) analyses for the different modalities. NiftyReg software was used to estimate the rigid‐body registrations between each individual's T1‐weighted and PET/dMRI images using a symmetric block‐matching approach,^16^ with the resulting transform used to resample the ROIs using nearest‐neighbor interpolation. GIFv3 was also used for estimation of total intracranial volume (TIV). dMRI pre‐processing included motion, eddy current, and susceptibility correction.^13^ The DTI model was fitted to the *b* = 700 shell and NODDI model fit after combining the *b* = 700 and *b* = 2000 shells. NODDI measures were weighted based on the tissue fraction (from the NODDI model) of each voxel to reduce estimation bias.^17^ All analyses were performed in native space.

All Aβ‐PET and tau‐PET images were included in analyses. Two individuals’ dMRI were significantly degraded by motion artifact and so were excluded. To limit multiple comparisons, image analyses were restricted to two composite cortical ROIs: (1) an MTL ROI (comprising hippocampus, parahippocampal, and entorhinal cortices), and (2) a wider cortical composite ROI (comprising lateral and medial frontal, anterior, and posterior cingulate, lateral parietal, and lateral temporal regions).^18^

First, within the two ROIs specified, we used Pearson correlation coefficients to quantify associations between Aβ‐PET SUVR and tau SUVR, cortical volume, MD, NDI, and ODI. We then did the same for associations between tau‐PET and individual MRI metrics.

Linear regression then assessed whether associations remained after adjustment for cortical volume. Diagnostic plots for each model confirmed the normality of residuals. Cortical volume measures were corrected for TIV. A statistical significance threshold of *p* < 0.05 was used throughout. No correction was made for multiple comparisons.

## 3 RESULTS

Participant demographics and clinical details are outlined in Table 1.

**TABLE 1:** Demographics and clinical/cognitive details of the study participants

*N*	14
Age (SD)	65.2 (4.9)
Sex (m/f)	6/8 (of those with dMRI is 6/6)
Clinical phenotype (typical AD/PCA)	7/7
Disease duration (SD)	8.0 (3.8)
ACE III (SD)	50.6 (13.5)

Abbreviations: ACE, Addenbrooks Cognitive Examination; PCA, posterior cortical atrophy; SD, standard deviation.

All 14 participants were Aβ PET positive, confirming underlying AD pathology. There was no evidence of a significant association between Aβ and tau SUVRs, either in the MTL or the cortical composite. In addition, there was no association between Aβ SUVR and any of the MRI metrics.

**FIGURE 1:**
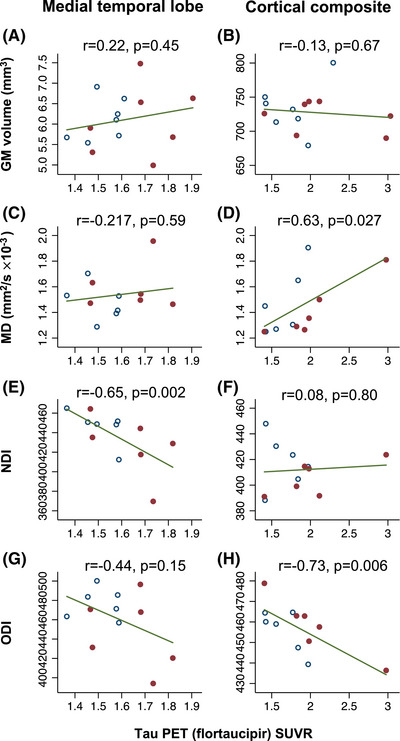
Associations between MRI imaging metrics and tau deposition. Scatter plots of different MRI imaging metrics (x‐axes) against cortical tau deposition as measured by Flortaucipir SUVR. The left column of plots presents data for the MTL meta‐ROI, and the right for the whole cortical composite. Pearson's r and *p*‐values are included for each association. Solid dots represent individuals with typical AD, with non‐solid dots representing posterior cortical atrophy. AD, Alzheimer's disease; GM, gray matter; MD, mean diffusivity; MRI, magnetic resonance imaging; MTL, medial temporal lobe; NDI, neurite density index; ODI, orientation dispersion index; SUVR, standardized uptake value ratio.

There was no significant association between tau SUVR and cortical volume in either ROI (Figure 1). In the MTL, there was a significant negative association between NDI and tau PET SUVR (Pearson's r = −0.65, *p* = 0.002), but not between MD and tau or ODI and tau. In the cortical composite, there was a significant association between MD and tau PET SUVR (r = 0.63, *p* = 0.027) and between ODI and tau (r = −0.733, *p* = 0.007) but not between NDI and tau (Figure 1). For associations between dMRI and cortical tau (Figure 1D, F and H), one possible outlier was identified with higher tau binding. On review, no technical reason was identified to omit the scan, and when repeating analyses excluding this individual the general pattern of associations persisted, remaining statistically significant for MTL NDI and tau, and for composite ODI and tau, but becoming no longer significant (*p* = 0.17) for composite MD and tau.

Linear regression, adjusting for regional TIV‐corrected cortical volume, confirmed a significant negative association between NDI and tau PET SUVR in the MTL (β = −0.14, partial R^2^ = 0.56, *p* = 0.008). An association between MTL ODI and tau (β = −0.13, partial R^2^ = 0.66, *p* = 0.002) was also seen, but not between MTL MD and tau. In the cortical composite, after adjusting for volume, there was a significant association between ODI and tau (β = −0.17, R^2^ = 0.43, *p* = 0.030) but not between NDI and tau or MD and tau.

## 4 DISCUSSION

In this study of individuals with young onset AD, using amyloid PET and tau PET alongside NODDI, we found higher tau deposition in the MTL to be associated with lower neurite density, a finding that persisted after correction for regional volume. This in vivo finding is consistent with there being a direct association at the microstructural level between focal tau pathology and focal loss of neurites, with implications for our understanding of the mechanisms underpinning focal neuronal loss in neurodegenerative disease.

There was no relationship between regional Aβ PET tracer uptake and either tau PET or any MRI measure. This was not unexpected given that the participants were at a disease stage when it would be expected that amyloid deposition had plateaued, and is consistent with multiple studies that demonstrated Aβ to not be tightly topographically coupled with either tau or neurodegeneration.^2^, ^19^

Higher tau load was also associated with lower ODI in the cortex; and in the MTL when volume was accounted for. Lower ODI is thought to reflect a decreased organizational complexity of neurites. Dendrites are the predominant neurite within cortical gray matter, and, therefore, are thought to be the main contributor to cortical ODI and NDI measures. These findings would be consistent with focal tau deposition leading to dendritic degeneration or pruning, building on previous imaging work to clarify the specific link between tau and microstructure, while also replicating in vivo what has been observed in post‐mortem histopathology.^3^, ^20^, ^21^ The fact that ODI changes were generalized throughout the cortex while NDI associations were seen only in the MTL, a region typically affected early in AD, is consistent with tau deposition leading first to pruning, and then to frank neurite density changes.

Our findings are also consistent in part with a previous study of NODDI and CSF biomarkers of AD pathology (which do not provide anatomic information), which found that alterations in Aβ alone were not associated with lower NDI but alterations in both p‐tau and Aβ in combination were.^22^ Of interest, however, the study did not find an association between AD CSF biomarkers and ODI, contrary to the associations we observe between ODI and tau‐PET.

Our results suggest that NODDI measures are more closely associated with AD tau pathology than either volume or DTI measures are. A study that used both NODDI and DTI in a mouse model of AD found that cortical NDI correlated with histological measurements of hyperphosphorylated tau, whereas other metrics including MD did not.^23^ Combined with our results, this raises the possibility that NODDI metrics, derived from standard 3T MRI multi‐shell acquisitions, may be able to provide indirect measurement of underlying tau without the expense and radiation exposure of a PET scan. If validated, such a tool would have significant implications for clinical practice, research, and therapeutic trials.

This study has a number of limitations. The cohort is small, and the results should, therefore, be interpreted with a degree of caution. Replication in larger AD patient groups will be valuable. The data presented are cross‐sectional only, and future longitudinal studies will be needed to confirm the temporal relationship between cortical tau and changes in NODDI metrics. We included patients with young onset disease, a substantial proportion of whom had an atypical, PCA phenotype where medial temporal loss typically occurs later in the disease; however, despite this we still saw a relationship between MTL NDI and tau deposition.

In conclusion, our findings demonstrate, for the first time, a close association between focal tau and in vivo measures of both dendritic complexity and dendritic density in AD. The results support the hypothesis that microstructural cortical neuronal changes are mediated by focal tau deposition. Multi‐compartmental modeling of multi‐shell dMRI, using approaches such as NODDI,^8^ has the potential to provide unique information on different aspects of microstructural degeneration, with additional sensitivity and specificity to degenerative changes compared with conventional imaging measures, and the potential to provide information pertaining to underlying tau deposition.

## CONFLICTS OF INTEREST STATEMENT

Philip S. J. Weston, William Coath, Matthew J. Harris, Ian B. Malone, John Dickson, Franklin I. Aigbirhio, David M. Cash, and Hui Zhang report no conflicts of interest. Jonathan M. Schott has received research funding and PET tracer from AVID Radiopharmaceuticals (a wholly owned subsidiary of Eli Lilly) and Alliance Medical; has consulted for Roche, Eli Lilly, Biogen, Merck, and GE; and received royalties from Oxford University Press and Henry Stewart Talks. He is Chief Medical Officer for Alzheimer's Research UK and Medical Advisor to UK Dementia Research Institute. Author disclosures are available in the supporting information.

## CONSENT STATEMENT

All participants provided written informed consent.

## Supporting information

Supporting Information
